# Professional Intercourse

**Published:** 1859-06

**Authors:** Daniel F. Wright


*Professional Intercourse. A Valedictory Address to the Graduating Class of Shelby Medical College, Nashville: Session of* 1858-9. By DANIEL F. WRIGHT, M. D.

*Gentlemen of the Graduating Class, and Fellow-Citizens:*—The event which we are here this night to witness is, to those of you who are principally concerned in it, beyond a doubt the most momentous of their lives; and to all of us (else why are we here?) there must be something of interest in it, something in which we are more or less directly, individually concerned—concerned, indeed, in different ways, and with a difference in the intensity of our feelings. Thus, we who have been actively engaged in the instruction and training of these young gentlemen, and thus brought into close and frequent personal intercourse with them, naturally feel a nearer and directer concern in the transaction than those who are personally strangers to them; and again, those, if there be any such among the general spectators, who are connected by the ties of kindred or friendship with our young friends, of course have their special interest in the occasion; but I am bound to assume that *all* who have taken sufficient interest in the matter to be here at all, have sympathies connected with it in some way or other, which it is my duty to find out, if possible, and appeal to.

And, first of all, young gentlemen, I am called upon to address myself to you. I think I can with less difficulty in your case than in that of others, conceive what are the feelings with which you are present here tonight, and therefore have a better chance of adapting my words to your present mental attitude. For, gentlemen, I have myself here now stood in the position you now occupy, and occasions like the present always bring very forcibly to my mind the conflict of feelings with which I came forward to receive in public the document which was to authorize me to take my position as a regular member of the medical profession.

Being, then, delegated by my colleagues, the Faculty of Shelby Medical College, to pronounce to you their farewell, to declare to you their good wishes, and to give you such parting counsel as may be remembered to good effect when you shall be scattered abroad in the respective fields of your professional calling, I enter on my task with what ability I may.

And first, for our good wishes.

If the tongue could speak as fervently as the heart can feel, we should here have no difficulty; but alas, speech is at any time but a poor exponent of feeling, and those whose feelings are the most deeply seated, are seldom the men who are the most ready at coining their emotions into words; and for myself, especially, I have, as you know, spent the greater part of my life as a student, secluded from those stirring scenes and jarring interests which most promote and develop the faculty of fluent utterance. I am no orator—and indeed I do not feel disposed, if I were able, at this moment to indulge in flights of oratory—I can neither look upon myself as an orator, nor on you as an audience; I would rather speak as friend to friend. Let me say, then, that one way or another I have been occupied in teaching for the last twenty years of my life; that in devoting myself to this duty, and to that of qualifying myself well for it, I have to a great extent lived apart from society, and that with me the sympathies and affections which are usually extended over family, friends, associates, etc., are concentrated upon those whom I have approached though their interest in the topics embraced in my instructions. When, after years of separation, I see a pupil in whose mind I may have been fortunate enough to kindle the first spark of enthusiasm in regard to some intellectual pursuit by which he has already achieved something of reputation or advancement, then, in the hearty hand-shake, and the brightening eye, with which old times are remembered, then comes my reward; that is to me in the place of personal fame, of kindred, friends, society, of every thing else which I have successively relinquished in the pursuit of science and literature; and I rejoice to say that such meetings are not rare; that again and again, where I least expect it, I fall in with an old pupil, who has soared his first flight in political life, and remembers the first kindling of his young ambition while studying with me the wisdom and eloquence of ancient Greece; or one who has made scientific attainment the road to profitable occupation, and dates his commencing progress from the trigonometry and chemistry of my school-room; or, last of all, some promising young physician who has instituted a new and successful treatment for the endemic diseases of his neighborhood, the first hint of which he is partial enough to recognize in some induction of my lecture-room; and I, who have but few social pleasures, you may judge am pleased then.—Yes, gentlemen, the peculiar sympathy which exists between a zealous teacher and an eager student is one which I am never afraid to intrust to time, for sound instruction is never so highly appreciated at the time it is given as when it has borne fruit after its own kind, and in a kindly soil increased its sixty-fold or its hundred-fold. *Thus*, gentlemen, may I hereafter meet many a one of you: you may not find in me the honeyed phrase and the fluent courtesy of the man of society, but you will find a hearty welcome, and a lively interest in your prosperity. I could say no more than this if I had the eloquence of a Cicero, and I would not say this either if I did not feel it from the bottom of my heart.

But I am here to give you counsel as well as good wishes, and with this view I have thought of some of the relations in life, some of the professional and personal trials in reference to which a little advice may not be unwelcome from one who has gone through it all before you. And I think that nothing in your future life will be likely so soon to render counsel desirable as that which consists in your newly established relations to the medical profession. Your responsibilities as regards its honor and interest, your conduct as regards its members, will from this day be among the weightiest subjects which can occupy your consideration.

Your bearing, then, towards your confrères, cannot be made the subject of minute and specific rules or, if it could, they would be much too numerous to be detailed on such an occasion as this. Definite rules, however, are not so important as an enlightened and liberal state of feeling between you and them. Let us consider what that feeling is likely to be—what it ought to be.

For some years the physicians with whom you will be brought into association will be, for the most part, your seniors—certainly none of them at present your juniors, Now, unfortunately, the relations between old physicians and young ones are seldom so cordial and friendly as they might be—as they ought to be. An alliance mutually very advantageous might and ought to exist; an antagonism mutually very injurious frequently does exist. I do not think the fault lies entirely on either side. Each thinks the other in the wrong, of course; the old man thinks the young one jealous of his practice, the novice thinks the old man jealous of his talents.

To commence with the young man, who, I imagine, will be rather the more interesting subject with the present audience. Every young physician who is worth the ink that is shed upon his diploma, starts in practice with the conviction that he is going to bring about a revolution in medicine He can distinguish between valvular disease of the right and left sides of the heart; he is acquainted with the virtue of *veratrum viride*, or *cannabis indica*, or some other hobby which he is going to mouut and ride over all obstacles to the highest distinction he can practice the radical cure of hernia; he can amputate at the shoulderjoint in four seconds—at least he has seen it done, and he thinks he could do it himself; he is going to put an end to the fumbling practice of the old doctors—no room for old fogies when he comes along: for this is another thing that young doctors are always predestined to do—they are going to demolish the old fogies.

Alas, that the days should have gone by forever when those were my own sentiments! for I, too, gentlemen, commenced my professional life, the would-be hero of a medical revolution. That revolution has not yet occurred. I have not much damaged the practice of the old fogies; there are a few who continue to practice that were included in that category when I got my diploma. On the contrary, there are certain papers upon the pathology and treatment of typhoid fever writtten by me in those happy days, which where to have paved the way towards the revolution aforesaid, but which, whenever I now fall in with them, I ruthlessly destroy, for fear any one in whose good opinion I desire to stand, should know that I ever wrote anything so conceited, shallow, and erroneous.

To return to our subject, however. That Class of physicians who are thus lightly spoken of as old forgies, are not generally very favorably disposed towards their younger competitors. Having spent thier lives in the practical study of disease, in resistance to its attacks, they are not likely to feel flattered with the unmistakable hints which my modest young revolutionists throw out, that their time is come, their maxims of treatment are obsolete, their diagnosis vicious, their practice inefficient. And thus, as I commenced with saying, we have in medicine, as in all other things, the same old contest between progress and conservatism—the prudence of age and the enthusiasm of youth. Let us not suppose that either of the parties to this controversy are wholly in the right, or wholly in the wrong, but rather (as it is desirable to moderate the intensity with which it is conducted) let us consider whether both parties may not do something towards instituting a better understanding.

As I shall have much more to say to the young friends than to the older practitioners, I will get through with the little I have to say to the latter first.

I commence, then, with addressing that venerable body, *Old Fogies*. I need not say that I do so with the most profound respect, for I find that old Time is gradually classing me with that estimable fraternity myself. I put it therefore to you, gentlemen, Is it necessary, is it desirable that we should be in a state of chronic irritation with the younger men? are we altogether free from blame when this gets to be the case? Let us suppose a case: An older physician is called in consultation where one of our novices is the attending physician.—What is his conduct? We know what, according to the courtesies of the profession, and the rules acknowledged by all of us, it ought to be. We know the delicate regard for the feelings of the attending physician, the subordination of individual pride and interest to the honor of the profession, which is called for by all the standards of professional ethics by which we profess to be guided; but do we always act upon the principles whose validity we recognize? I think not. I think that there are few physicians now listening to me who cannot recall some instances in which, when they commenced the practice of medicine, they have not in their earlier consultations been treated by older physicians in a manner very different from what the spirit of our ethics calls for. Thus in the first place we will suppose that the diagnosis or the treatment of the young physician bears some marks of inexperience: of course the maturer experience of the consulting physician should correct these errors; but is this always done with all the forbearance and consideration which we all admit is right? is the proper reserve exhibited in speaking of the previous treatment? Is the mantle of charity thrown over the possible errors of the younger practitioner? I must say that as far as my experience goes, while I have had much to be grateful for in the way of counsel and support in difficult circumstances from my seniors, I have meet with much that was done in a very opposite spirit. I have se n many who would on the first suspicion of an error, so hold it up and blazon it as to get the case at once transferred to their own hands. But you will say that this is unprofessional, that the *Code of Ethics* provides for the sanctity of consultations. Alas, it illustrates how powerless codes of ethics are in protecting the struggling novice against the established practitioner, unless the latter has a code of ethics deeply impressed in his own heart.

But we will take milder instances. The old practioner, like the veteran in other matters, is apt to be *"laudator temporis acti"* a believer in the past, a stickler for established practices. Velpeau denies the diagnostic value of the microscope; many respectable old gentlemen still hold out against auscultation as a humbug; and, as a general thing, you will find that if in a consultation a younger man proposes something which is new to the elder physician, his first impulse is to oppose it, make light of it. My venerable friends, this is a bad sign. When you find yourself doing this, be assured you are growing old; watch yourselves—you will find that you totter over your saddle bags, that your lancet makes two or three plunges to the right and left before it plumps the centre of the vein: take care, you are digging your own graves. There are some physicians in whom this symptom develops itself alarmingly early; it commences from the day they get their diplomas; they shut their books then, they open no others; half a dozen pet operations, a few stereotyped prescriptions circumscribe their practice, and before their beards are well established on their chins, they degenerate into the premature decrepitude of young fogyism. I venerate and love the old physician who welcomes the boys from college, and asks them the new items, turns over their books, handles their new instruments, takes them by the hand, gives them his advice, and (which they like bett.r) asks them for information in return.

You know the old Anacreontic we learned at college: "I love the cheery old man, I love the dancing youth; but when the old man dances with the boys, his hair may be gray, but his heart is young."

My venerable friends, I don't want to set you dancing, but I do contend that the truly great men in all departments of greatness have been those who knew how to combine with the experience of age the ardor, the impressibility, the freshness of youth, who have continued young in their capacity for new ideas, while they grew old in the accumulation of wisdom; these have been the men whose names are recorded with veneration in the annals of art and science with gratitude in the records of a nation's prosperity and advancement.

No, I take it back! I am not an old fogy; I don't intend to be one forty years hence. No man need grow old who will keep his heart and intellect young—gray hairs are nothing—wrinkles are nothing; and, my fellow-practioners, I advise you, keep on good terms with the boys; you can get as much good from them as they from you, if it be only in the way of helping to keep the wrinkles from your heart, the gray hairs from striking in upon the brain.

But the lapse of time reminds me that it is my main duty to address myself to the junior portion of the profession, to young medicine as represented in the person of the graduates of this night.

You, too, gentlemen, are interested in the harmony of the profession; you have everything to gain by the establishment of a cordial understanding with the rest of the profession; you have duties to render towards sustaining it; you have your share of the blame if it be broken.

I fear it cannot be denied that much of the suspicion, distrust, and hauteur with which old practitioners are apt to treat the recruits of the profession, is provoked and excused by the self-sufficiency and aggressive pertinacity with which their ideas and claims are sometime spushed upon the notice of their elders. I will assume that when you arrive at what is to be the field of your professional duties, you will be in possession of several recent improvements which have not hitherto been put in practice there. It has been the duty of seven men for the last two years to rake together for your instruction all that the recent discoveries and advances of medicine have furnished in the way of available knowledge, and it has been your duty to learn all that they could teach you; and there must be some strange neglect of duty on the one side or the other, if you have not learned something which men engaged in the laborious and engrossing duties of practice have not had time to keep up with. But is it necessary for you—would it be necessary for you if you were in possession of double that vast amount of wisdom which has so wonderfully found room within your swelling brains, to so throw it at people as to offend them, to blow your trumpet so loudly and incessantly as to render yourselves and your trumpets a nuisance?

To be more serious, you must be conscious that a great deal of what you at present know cannot but be crudely known; that until repeatedly reduced to practice, and its effects observed, it is theory rather than knowledge with you; in short, that you are at present deficient in experience; and although nobody despises more than I do that false experience which pursues the same unthinking routine at the bedside year by year, learning nothing, still no knowledge is truly assimilated and incorporated into the mind until it has been used, and so both amplified and corrected.

Yes, there must already have insinuated itself into your minds, side by side with the exultation with which you assume your new position as doctors of medicine, a surmise, a suspicion that you have grounds for anxiety as well as triumph—that you have incurred a heavy load of responsibility, as well as attained to a position of high trust and privilege. At least I hope so, for if not, the feeling will come upon you the more overwhelmingly and crushingly when it does come: and come it will, sooner or later, be well assured. Yes, the stoutest-hearted among you has soon to encounter scenes which will make even his stout heart quail. It is no small matter, however strong your nerves may be, to be summoned where a valued citizen, a beloved husband and father, has been surprised in the death-grapple with some terrible malady, and all eyes are looking to you: the powers of life, the throes of death are engaged in mortal conflict in that one convulsed and fevered body: mysterious and inscrutable processes, some tending towards dissolution, others towards preservation, are going on there; for mysterious and inscrutable are they still, spite of the giant labors and brilliant achievements of modern science. And there you stand, all eyes upon you—you, who are supposed to hold in your hands the clue for the solution of all these mysteries, the enchanter's wand which is to still the conflict. And you are in interrogated—not in words, for hearts are too full to speak, but by speechless questioning eyes. How is it with him? Must he die? Must we lose our father, husband, friend? Can you save him, Doctor? Will you? Ah, my friends, too much complaint has been made by medical men, of late, about the want of confidence in medicine, but my experience is, that the physician, when once seriously engaged in practice, suffers more from the too much than the too little that men expect and demand from it.—But to return to our bedside, do you not now begin to suspect that you would derive support and satisfaction from a conference with some physician who has spent life in similar scenes, in observing and treating similar conditions? You will feel it, my friends; you *will* be forced to conclude that the much you know, or think you know, is as nothing compared to the infinity of which you know nothing; that even what you know does not occur to you as promptly as you imagined it would; that diseases you thought you had thoroughly studied, seem to you almost as new as if you had never heard of them; and if by this time your emotions are not of a most painful character, you are more than man—no, you are less than man, for the heart which does not feel on such occasions is not superhuman, it is brutal.

Yes, if you have human hearts you will gladly seek to share the burdens of your responsibility with some one of graver experience; and you will now begin to perceive your error, if, by arrogance, conceit, and flippancy, you have repelled from yourself that sympathy which now you need. You will remember these admonitions of mine, however homely they may be, when the time comes, though they may be forgotten very soon after they are delivered. It is the interest of both of you, I assure you, that you should thoroughly work together. Even in a pecuniary point of view it is unwise to be at variance.—Look round among the practitioners in any community you are acquainted with, and who is the thriving man among the established physicians? I will answer before I know him, he whom the young men like to call in consultation when they are in doubt or trouble.—Who is the rising one among the young practitioners? Why, he whom the established doctors like to call to their assistance, when they have a delicate operation calling for trustworthy assistance; and believe me, if you manifest at all a commendable skill, and a moderate degree of respect and courtesy towards your senior, the old man would rather have you at his elbow than his more established rival: there is a good deal of human nature even in old country doctors. What is the lesson, then, to be learned from all this? Simply the golden rule of manners all the world over, *Learn to be respectful without ceasing to be self-respectful.* I give it to you as the key-note of all conclusions relating to medical manners.

But a few words to those who, unconnected with the medical profession, have nevertheless thought the transactions of this night worthy their recognition and attention. We have a right to assume that you are interested in it—and you ought to be—for who is not concerned in the selection and training of those who are to aid and relieve them when pain and suffering and the fear of death are upon them? At a transient glance, one would be disposed to imagine that the public were more interested in every evanescent *ism*, in every boastful and noisy charlatan, than in the regular medical profession, and its professors and practitioners, and their transactions; but it is not so. It is not the men who make the most noise in the world, who get themselves blazoned in the newspapers, and parade their miraculous nothings at the corners of the streets, that most possess or most deserve the confidence of people; but those who perseveringly and conscientiously, day by day, and night by night, pursue their noiseless labors, trusting for reputation to what they do, to what others say of them, and not to what they say of themselves. These are the men who, with the great body of right judging people, are received as their friends, their confidants, their benefactors. And the great body of men generally do judge right. I care little for catching the notice of those who are always on the watch for flaring and spacious novelties; they are seldom worth catching. I would much rather create a favorable impression with those sober, thoughtful men who are repelled rather than attracted by self-sufficient parade. To such, then, I now address myself, and request their attention to the true nature of the transaction just witnessed.

Ladies and gentlemen, we are here to recommend to your favorable acceptance the young gentlemen whose names you have just heard, and whom you are now called upon to receive and greet as *Doctors of Medicine.* We challenge on their behalf your confidence, not in consideration of any boastful assertions they make of themselves, not in virtue of testimonials cajoled from those ignorant of them and of the true value of medical procedure, but upon the strength of our having subjected them to a rigorous scrutiny in regard to their attainments. And I assure you here, and now, that, in spite of vulgar misconceptions upon the subject, our scrutiny has been no child's play. I for one have never known any thing so seriously discussed or transacted as the proceedings of the last few days; and consider it as affection if you will, I will nevertheless say it: if you had witnessed the last consultation of yesterday, in regard to who should be found worthy of our diplomas, you would yourselves have been deeply impressed with the solemnity, and profound—I might say painful—sense of responsibility with which it was done.

No, my friends, let popular derision make what it will of it, it is no light matter with us to invest our students with the title and position they have now assumed: they have earned it, or they could never have received it.

Ladies, I cannot refrain from the assertion that you, more than all others, are concerned in this transaction. When sickness has spread the bed of suffering in a household, and the terrors of anticipated bereavement have possessed its trembling inmates, thither the fine and beautiful instincts of your sex always call you, so that it is an unfailing rule that where sorrow is, there, also, is woman, its consoling angel; and in that darkened chamber, the physician alone, of all men, is privileged to be your companion, the trusted partner and aider of your charitable toils, your holy vigils, your angelic ministrations. Is it not, then, of the last importance to you that he who is associated with you, when all the finest sensibilities are strung up to the highest pitch, where any thing discordant with them would be agony, he who must then be either a valued friend or an intolerable intruder, should be not only prudent and able, but refined and delicate, not only an accomplished physician, but a finished gentleman?

Such, ladies, are the physicians whom it is our aim to produce.—The enterprise in which, as the Faculty of Shelby Medical College, we are for life engaged, is that of annually sending forth a body of young physicians who shall be carefully trained, not only scientifically, but morally, for the great responsibilities, the delicate and self-sacrificing duties which await them. Give them, ladies, the cheering encouragement of your smiles and favors at their starting: let them go forth with the inspiring conviction of your approbation, your good wishes, your fervent prayers in their behalf.

Finally, gentlemen, I must say briefly, because I know not how to say it eloquently—to say it as I feel it—*Farewell.* You have made friends in us, your instructors, who have that interest in you that no one else can have With you it rests whether the labors we are undergoing for the moral and intellectual elevation of the profession shall prosper or not. We can never cease, therefore, to be interested about you. We can only say, then, in the simple language of friends, who shake hands at parting, "Come and see us often;" if you can't, let us hear from you; never be afraid to ask us for advice or instruction or assistance. In one word, God bless you; go and prosper; your success is our success, your honor is our honor.—*Nashville Medical Record.*

